# Reliability, Consistency and Temporal Stability of Alberta Infant Motor Scale in Serbian Infants

**DOI:** 10.3390/children7030016

**Published:** 2020-03-02

**Authors:** Milan Lackovic, Dejan Nikolic, Dejan Filimonovic, Ivana Petronic, Sladjana Mihajlovic, Zoran Golubovic, Polina Pavicevic, Dragana Cirovic

**Affiliations:** 1 Clinical Hospital Center “Dr Dragiša Mišović”, 11000 Belgrade, Serbia; lackovic011@gmail.com (M.L.); drsladjanamihajlovic@gmail.com (S.M.); 2 Faculty of Medicine, University of Belgrade, 11000 Belgrade, Serbia; dejan.filimonovic@gmail.com (D.F.); ivana.pm@live.com (I.P.); dr.dejan.nikolic@outlook.com (Z.G.); pzmbov@yahoo.com (P.P.); 3 Physical Medicine and Rehabilitation Department, University Children’s Hospital, 11000 Belgrade, Serbia; 4 High-Risk Pregnancy Unit, Obstetrics/Gynecology Clinic “Narodni font”, 11000 Belgrade, Serbia; 5 Pediatric Surgery Department, University Children’s Hospital, 11000 Belgrade, Serbia; 6 Radiology Department, University Children’s Hospital, 11000 Belgrade, Serbia

**Keywords:** Alberta Infant Motor Scale, reliability, consistency, temporal stability, Serbian version

## Abstract

Our study aimed to analyze the reliability, consistency, and temporal stability of the Alberta Infant Motor Scale (AIMS) in Serbian infants. Additionally, we aimed to present a percentile distribution of AIMS in the tested population. The prospective study included 60 infants that were divided into three age groups: 0–3 months, 4–7 months, and 8–14 months. The Serbian version of AIMS was tested by two raters on two different occasions (test/retest) with a five day period between tests. The observed inter-rater reliability (intraclass correlation coefficient (ICC)) was more than 0.75 for all AIMS scores, except for standing (ICC 0.655 = moderate) in the age group of 4–7 months on retest between raters. The observed intra-rater reliability (ICC) was more than 0.75 for all AIMS scores except standing (ICC 0.655 = moderate) in the age group 4–7 months in test–retest for Rater One, and for sitting (ICC 0.671 = moderate) and standing (ICC 0.725 = moderate) in the age group between 0–3 months on test–retest for Rater Two. The Serbian version of AIMS was shown to have high consistency and high reliability with good to high temporal stability. Thus, it can be used in the evaluation of infants’ motor development in Serbia.

## 1. Introduction

The complexity of the pediatric population within the first year of life refers to the fact that motoric development is rapid and might be influenced by various degrees by environmental, biological, and social factors [1]. The necessity of proper and reliable estimation of motor development in the first year of life is stressed by an increase in the survival rate of more than 85% in very preterm infants [2]. Furthermore, motor, cognitive, and behavioral impairments were found in more than 50% in this group of infants [2].

In previous reports, it was stressed that numerous environmental as well as biological factors could be responsible for developmental delay and challenged motor skills in children [3,4,5]. This is of great importance since the processes that govern motor development might be influenced before and after birth.

Discrepancy between functional and chronological age could classify the degree of developmental delay as mild, moderate, and severe [6]. Therefore, there is a great need not only for detecting the presence of developmental delay, but also the degree for proper and optimal inclusion in the treatment.

Considering major standardized tests, two types of measures are used: norm-referenced and criterion-referenced [1,7]. Motor handicap identification in children could be identified by norm-referenced tests, while interventional programs and their effectiveness by criterion-referenced tests [7].

Alberta Infant Motor Scale (AIMS) is a norm-referenced, observational, and performance-based measure [8]. It has been shown to have high sensitivity and specificity in motor deficit detection [9]. Furthermore, AIMS is used in the evaluation of functional capacities, spontaneous movement activities, and quality of movement [8,9].

Our study aimed to analyze the reliability, consistency, and temporal stability of AIMS in Serbian infants. Additionally, we aimed to present the percentile distribution of AIMS in the tested population.

## 2. Methods

### 2.1. Study Group

The prospective study included 60 infants that were referred to University Children’s Hospital (UCH) for the evaluation of motor development. Participants from the neonatology and pediatric rehabilitation departments of UCH participated in the study, and were recruited on the merit of being at risk for motoric delay by having one or more risk factors. The inclusion criteria were age between 0–14 months and being at risk for motor delay. The risk factors for the motor delay were birth weight below 1500 g, gestational age below 32 weeks, Apgar score lower than 7 at 1 and 5 min, central nervous system infection, intraventricular hemorrhage, and chronic lung disease [10,11]. Both genders were included. The participants were divided into three age groups: 0–3 months, 4–7 months, and 8–14 months [10].

### 2.2. Translation Process

We used the forward–backward method for translation of the AIMS from English into Serbian [12,13]. The translation process for AIMS was done according to the principles of a framework for translation and cultural adaptation [14]. Two board-certified physiatrists with clinical experience of five and more years were engaged in the translation process of the AIMS into Serbian, generating two versions. Under the supervision of the translator, after comparisons, these versions were merged into the final forward translation. The translator who participated in the translation was fluent in English and Serbian. Furthermore, back-translation into English (backward translation) was done by another independent translator who was also fluent in English and Serbian. After consensus had been obtained between the discrepancies of the forward and backward translations, the probationary merged AIMS version was tested on ten infants that were selected from the database of the medical records in the pediatric rehabilitation medicine UCH ambulatory setting. Two infants per day from the five days ambulatory database in one week were chosen by chance. Parents or legal guardians were then contacted and invited to participate. The response rate was 100%. Since AIMS is an observational scale, no feedback was received, and after the expert panel meeting, the final translated version of AIMS was introduced.

### 2.3. Measuring Instruments

The AIMS has 58 items that are grouped into four subscales: pronation (21 items), supination (nine items), sitting (12 items), and standing (16 items) [15]. For every item, three main descriptors of motor performance are described: weight-bearing, posture, and antigravity movement [11].

Two board-certified physiatrists (Rater 1 and Rater 2) tested the AIMS on the pediatric population. In Serbia, board-certified physiatrists are specialists of physical medicine and rehabilitation, who can independently perform the functional assessment of patients. For the pediatric population, they perform examinations that also include the estimation of motor development in infants. Both raters had five or more years of experience in pediatric rehabilitation practice and neurodevelopment assessment in children, and did not have prior experience in the AIMS scoring. Raters were informed about administration, testing procedures, rating criteria, and scoring of the AIMS. They attended four hours of practical assessment over two days for implementation and scoring of the AIMS. Both raters initially tested 16 children with the AIMS on two occasions with five days between the test and retest. Eligible participants were selected from the database of the medical records in the pediatric rehabilitation medicine UCH ambulatory setting. Four infants per day from the four days ambulatory database in one week were chosen by chance. Parents or legal guardians were then contacted and invited to participate. These children were not included in the study analysis. After initial testing, 60 infants were recruited and tested by the Serbian version of the AIMS twice (test—initial; retest—five days after initial testing). To avoid potential bias and ensure independent scoring, two raters were not allowed to discuss the tested findings.

### 2.4. Statistical Analysis

The obtained results were presented as whole numbers (N), percents (%), mean values (MV) and standard deviation (SD). Differences in mean values between raters on both occasions as well as between the test and retest for every rater were analyzed by the Mann–Whitney U test. Total AIMS scores were also presented as percentiles (10^th^, 25^th^, 50^th^, 75^th^, and 90^th^ percentile) for both raters on both occasions.

The consistency of the AIMS scores between the test and retest for both raters and between raters was done by Cronbach’s alpha. A Cronbach’s alpha above 0.9 was deemed as excellent, 0.8–0.9 good, 0.7–0.8 acceptable, 0.6–0.7 questionable, and 0.5–0.6 poor internal consistency [16].

Inter-rater and intra-rater reliability was done by the intraclass correlation coefficient (ICC). Reliability according to the values of ICCs were grouped as: >0.90 = high, 0.75–0.90 = good, 0.50–0.75 = moderate, and <0.50 = poor [17]. Inter-rater reliability was additionally analyzed by Bland–Altman plots. Statistical significance was set at *p* < 0.05.

## 3. Results

In Table 1, the characteristics of the tested participants are presented.

There was excellent internal consistency between the test and retest for both raters (Cronbach’s alpha _Rater1_ for group 0–3 months = 0.941; 4–7 months = 0.998, and 8–14 months = 0.998; Cronbach’s alpha _Rater2_ for group 0–3 months = 0.937; 4–7 months = 0.997, and 8–14 months = 0.998) as well as between raters on the test for every age group (Cronbach’s alpha _Test(Raters1/2)_ for group 0–3 months = 0.940; 4–7 months = 0.998, and 8–14 months = 0.996).

In Table 2, the mean values of the AIMS scores are presented for every subgroup and as the total score. There is no difference in the mean values between different raters in every age group both on initial examination (*p* > 0.05) and at retest (*p* > 0.05) (Table 2). The highest scores were for the prone position in every age group, and lowest for the standing position.

Inter-rater and intra-rater reliability of the AIMS scores are presented in Table 3. The observed inter-rater reliability (ICC) was more than 0.75 for all AIMS scores (range from 0.782 for standing task in the age group between 4–7 months on test to 0.995 for the total in age group between 4–7 months on test), except for standing (0.655 = moderate) in the age group between 4–7 months on retest between raters (Table 3). Differences in the mean values between raters both for the test and retest were measured by the Mann–Whitney U test, and were non-significant (*p* > 0.05). The observed intra-rater reliability (ICC) was more than 0.75 for all AIMS scores (range from 0.782 for the standing task in the age group between 4–7 months for Rater 2 to 0.997 for the standing task in the age group between 8–14 months for Rater 1 and Rater 2, and in the total score for the age group between 4–7 months for Rater 1), except for standing (0.655 = moderate) in the age group of 4–7 months in the test–retest for Rater 1, and for sitting (0.671 = moderate) and standing (0.725 = moderate) in the age group between 0–3 months on the test–retest for Rater 2 (Table 3). Differences in the mean values for each rater between the test and retest were measured by the Mann–Whitney U test and were non-significant (*p* > 0.05).

**Figure 1:**
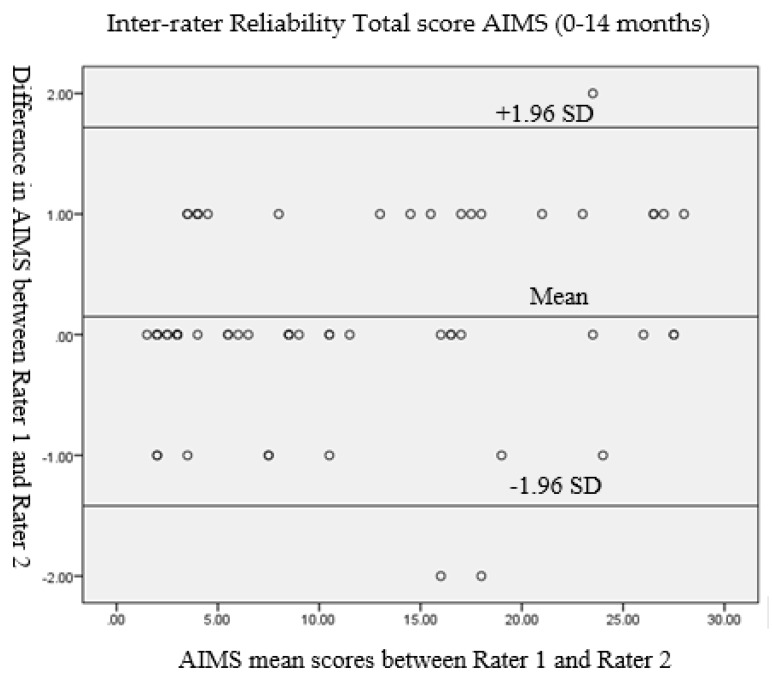
Bland–Altman plot of inter-rater reliability of the Alberta Infant Motor Scale (AIMS) total score between Rater 1 and Rater 2. SD: standard deviation.

Inter-rater reliability of the AIMS total score on the test for all tested infants between Raters 1 and 2 was presented and evaluated by the Bland–Altman analysis (Figure 1). For Rater 1 and Rater 2, the mean difference was 0.15, the SD of the differences was 0.80, the lower limit was −1.42 (for −1.96SD), and the upper limit was 1.72 (for +1.96 SD).

**Figure 2:**
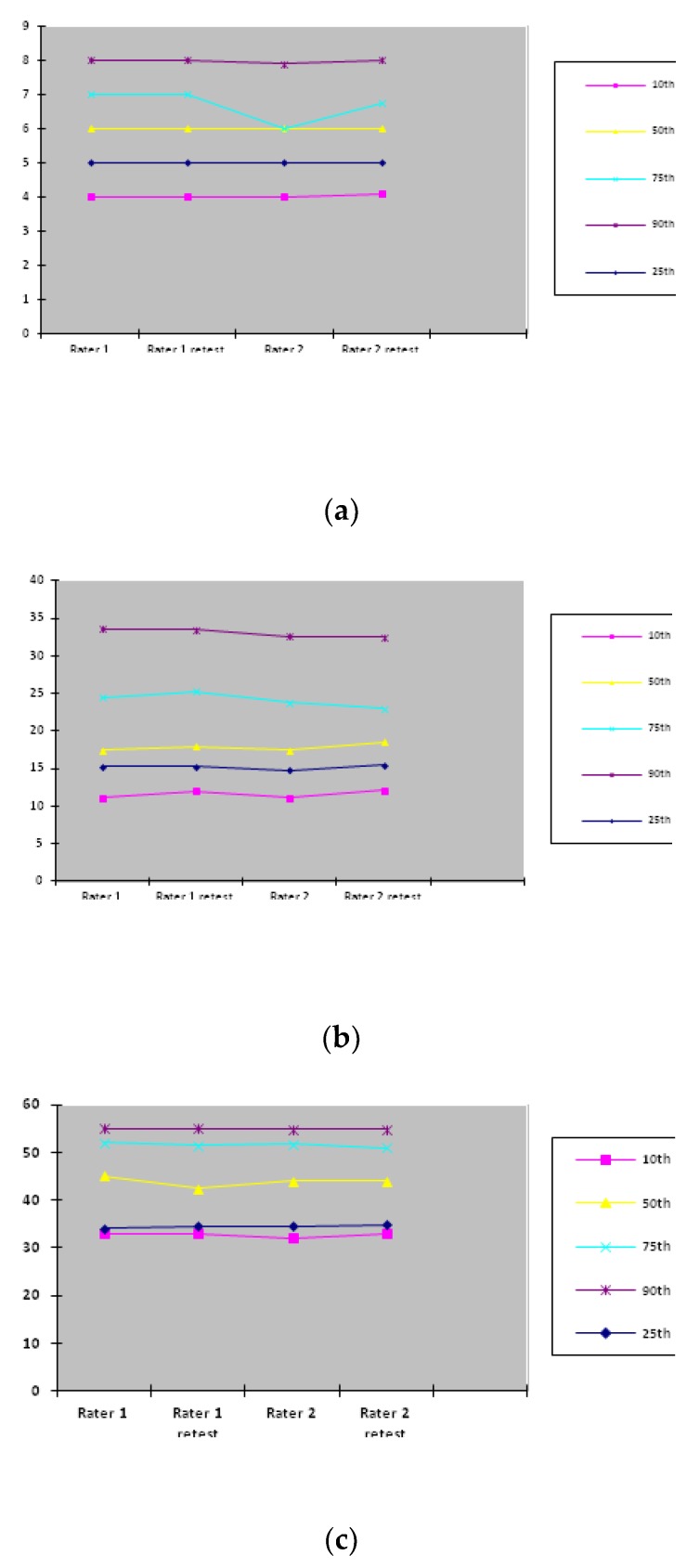
**The** AIMS score percentile distribution in different age groups: (**a**) 0–3 months age group; (**b**) 4–7 months age group; (**c**) 8–14 months age group.

In Figure 2, percentile distributions for each rater on both occasions in different age groups are presented. The most consistent percentile distributions for the total AIMS scores were for Rater 1 (test–retest) in the age group between 0–3 months and for Rater 2 (test–retest) in the age group between 8–14 months. Percentile distributions, especially for the 25^th^ and 90^th^ percentiles, did not show obvious temporal fluctuations in each age group.

## 4. Discussion

In this study, we aimed to translate and analyze the reliability, consistency, and temporal stability of the Serbian version of the AIMS on the Serbian language for the Serbian pediatric population. The final translated version of AIMS followed the forward–backward method for translation to prevent the possibility of biased translation from the different cultures [18]. The possible roles of cultural differences in different populations, particularly for the AIMS scores, were elaborated in the study by De Kegel et al. [19], where it was noticed that sleep position, playing time in prone or supine positions, and a sitting device might be associated with lower motor scores in Flemish infants versus the Canadian reference group between 1990–1992. However, another study on Greek infants showed a similar course of gross motor maturity during the first 18 months when compared to Canadian infants measured by the AIMS, with a few exceptions [20].

Our study demonstrated that the Serbian version of the AIMS had high internal consistency with Cronbach’s alpha values ranging between 0.937–0.998, implying the high homogeneity among variables.

Considering inter-rater reliability, there was strong agreement between the two raters for the total scores for each age group of the Serbian version of AIMS (ICC 0.887–0.995), and thus good to high reliability. Furthermore, the mean values of the scores for each subgroup and in total for each age group did not significantly differ between raters. This points to the fact that the results of inter-rater reliability in this version of AIMS could be considered as correct [21].

For the evaluation of the temporal stability of the Serbian version of AIMS, intra-rater reliability for the total scores in each age group was good to high (ICC 0.860–0.997) with non-significant differences between mean values for each subgroup and total of each age group. Furthermore, percentile distributions, particularly the 25^th^ and 90^th^ percentiles, did not show obvious temporal fluctuations in each age group. However, in infants, it was previously identified that percentile fluctuations of motor abilities might not necessarily be indicative of motor dysfunction [22]. Moreover, Darrah et al. suggested that for normally developing infants, the motor development rate is not stable [22]. We have presented the lower percentile rank values at the 10^th^ percentile, even though in the study by Darrah et al., it was suggested that for children of eight months and above, the 5^th^ percentile should be considered as the optimal value [23]. However, the sensitivity value for a group of infants at eight months is higher for the 10^th^ percentile than for the 5^th^ [23]. Therefore, this Serbian version of AIMS was shown to have satisfactory temporal stability.

For intra-rater reliability, in the age group between 0–3 months of life, the sitting and standing values were lower for Rater 2 (ICC 0.671; ICC 0.725, respectively), while for those between 4–7 months, the standing values were lower for Rater 1 (ICC 0.655) and Rater 2 (ICC 0.782). The lower values might be explained by the difficult assessment of younger infants in situations with poor cooperation. Our findings are, to a certain degree, in line with previous reports, particularly for standing components in the early infant period [17].

Proper evaluation of the motor development of infants, particularly those who are at an increased risk for a delay, is of great importance for an adequate and onetime inclusion of effective treatment with proper follow-up. It was stated that AIMS could potentially be useful in the detection of motor deficits in an early stage for high-risk infants [17]. Effective decision making and strategies for the good management of children with motor delay is still insufficient.

## 5. Study Limitations

There are several limitations to this study. Even though it was conducted at the referral center “University Children’s Hospital” with their highly skilled and educated personnel, the first limitation refers to the participation of one center (single-center), thus further studies for increased sensitivity are also advised in other centers in the country. Moreover, another limitation refers to the small number of participants that might affect the level of the study’s statistical power. Furthermore, children were not randomly selected, and no other established motor infant tests such as the Bayley Motor Scale or Peabody Gross Motor Scale have concurrently been performed on infants at risk for motor delay to support the findings related to AIMS.

## 6. Conclusions

The Serbian version of AIMS was shown to have high consistency and high reliability with good to high temporal stability. Thus, it can be used in the evaluation of infant motor development in Serbia.

## Figures and Tables

**Table 1:** Characteristics of the tested participants.

Parameters		Participants	
Gender N (%)	Male	34	(56.7)
	Female	26	(43.3)
Intraventricular Hemorrhage N (%)	Grade I-II	5	(8.3)
	Grade III-IV	1	(1.7)
Chronic lung disease N (%)		5	(8.3)
Apgar score MV ± SD (Range)	1 min	7.03 ± 1.63 (2–9)	
	5 min	8.87 ± 0.91 (6–10)	
Gestational age (weeks) MV ± SD (Range)		34.22 ± 3.15 (26–40)	
Birth weight (grams) MV ± SD (Range)		2097.70 ± 668.52 (620–4030)	

MV: mean values; SD: standard deviation.

**Table 2:** Alberta Infant Motor Scale score mean values in different age groups by Rater 1 and Rater 2.

Group	Rater 1 Test	Rater 2 Test	Rater 1 Retest	Rater 2 Retest
	MV ± SD	MV ± SD	MV ± SD	MV ± SD
0–3 months				
Prone	2.25 ± 0.91	2.20 ± 0.83	2.25 ± 0.97	2.30 ± 0.92
Supine	1.80 ± 0.83	1.75 ± 0.79	1.95 ± 0.89	1.85 ± 0.81
Sitting	1.30 ± 0.57	1.30 ± 0.47	1.40 ± 0.60	1.35 ± 0.49
Standing	0.40 ± 0.60	0.40 ± 0.60	0.50 ± 0.61	0.50 ± 0.61
Total	5.80 ± 1.44	5.65 ± 1.39	6.10 ± 1.65	6.00 ± 1.38
4–7 months				
Prone	8.30 ± 3.83	8.30 ± 3.64	8.45 ± 3.80	8.35 ± 3.53
Supine	5.00 ± 1.69	4.95 ± 1.64	4.85 ± 1.66	5.10 ± 1.55
Sitting	4.90 ± 1.68	4.85 ± 1.53	4.90 ± 1.68	4.95 ± 1.47
Standing	1.90 ± 0.31	1.85 ± 0.37	1.95 ± 0.22	1.90 ± 0.31
Total	20.10 ± 7.21	19.95 ± 6.85	20.15 ± 7.04	20.30 ± 6.45
8–14 months				
Prone	17.00 ± 2.25	16.90 ± 2.10	17.05 ± 2.14	16.80 ± 2.02
Supine	7.95 ± 1.00	7.95 ± 1.10	8.05 ± 1.05	8.00 ± 1.03
Sitting	11.15 ± 0.81	11.15 ± 0.88	11.15 ± 0.88	11.20 ± 0.83
Standing	7.85 ± 4.84	7.70 ± 4.75	8.00 ± 4.92	7.75 ± 4.72
Total	43.85 ± 8.73	43.70 ± 8.44	44.30 ± 8.73	43.75 ± 8.19

**Table 3:** Inter-rater and intra-rater reliability of the AIMS scores.

Group	Inter-Rater Reliability Rater 1/Rater 2 (Test)		Inter-Rater Reliability Rater 1/Rater 2 (Retest)		Intra-Rater Reliability Rater 1 (Test-Retest)		Intra-Rater Reliability Rater 2 (Test-Retest)	
	ICC	95% CI	ICC	95% CI	ICC	95% CI	ICC	95% CI
0–3 months								
Prone	0.901	0.769–0.960	0.859	0.679–0.942	0.943	0.862–0.977	0.936	0.846–0.974
Supine	0.962	0.908–0.985	0.862	0.689–0.943	0.900	0.758–0.960	0.922	0.815–0.968
Sitting	0.816	0.589–0.923	0.916	0.804–0.966	0.855	0.673–0.940	0.671	0.334–0.855
Standing	0.859	0.677–0.942	0.863	0.686–0.944	0.863	0.690–0.943	0.725	0.432–0.880
Total	0.887	0.741–0.953	0.913	0.796–0.965	0.876	0.707–0.950	0.860	0.643–0.945
4–7 months								
Prone	0.993	0.982–0.997	0.978	0.945–0.991	0.995	0.986–0.998	0.994	0.985–0.998
Supine	0.973	0.933–0.989	0.914	0.791–0.965	0.973	0.931–0.990	0.971	0.924–0.988
Sitting	0.971	0.929–0.988	0.930	0.832–0.972	0.982	0.956–0.993	0.956	0.893–0.982
Standing	0.782	0.533–0.907	0.655	0.317–0.846	0.655	0.317–0.846	0.782	0.533–0.907
Total	0.995	0.989–0.998	0.983	0.958–0.993	0.997	0.991–0.999	0.993	0.979–0.997
8–14 months								
Prone	0.979	0.948–0.992	0.948	0.870–0.979	0.984	0.961–0.994	0.965	0.914–0.986
Supine	0.955	0.889–0.982	0.836	0.632–0.932	0.952	0.885–0.981	0.933	0.841–0.973
Sitting	0.930	0.831–0.972	0.897	0.760–0.958	0.930	0.831–0.972	0.897	0.760–0.958
Standing	0.992	0.981–0.997	0.988	0.970–0.995	0.997	0.992–0.999	0.997	0.992–0.999
Total	0.993	0.982–0.997	0.987	0.965–0.995	0.994	0.983–0.998	0.995	0.988–0.998

ICC: intraclass correlation coefficient.
